# Endobronchial coils for emphysema: Dual mechanism of action on lobar residual volume reduction

**DOI:** 10.1111/resp.13816

**Published:** 2020-04-08

**Authors:** Jorine E. Hartman, Pallav L. Shah, Frank Sciurba, Felix J.F. Herth, Dirk‐Jan Slebos, RENEW Study Group, F.J.F. Herth, D. Gompelmann, M. Schuhmann, R. Eberhardt, D. Harzheim, B. Rump, D.J. Slebos, N. Ten Hacken, K. Klooster, J.E. Hartman, S. Augustijn, P.L Shah, C. Caneja, W. McNulty, J. Garner, G. Deslée, H. Vallerand, S. Dury, D. Gras, M. Verdier, C.H. Marquette, C. Sanfiorenzo, C. Clary, C. Leheron, J. Pradelli, S. Korzeniewski, P. Wolter, T. Arfi, F. Macone, M. Poudenx, S. Leroy, A. Guillemart, J. Griffonet, C. Strange, R. Argula, G. Silvestri, J.T. Huggins, N. Pastis, D. Woodford, L. Schwarz, D. Walker, G. Criner, J. Mamary, N. Marchetti, P. Desai, K. Shenoy, J.L. Garfield, J. Travaline, H. Criner, S. Srivastava‐Malhotra, V. Tauch, R. Maxfield, K. Brenner, W. Bulman, B.A. Whippo, P.A. Jellen, R. Kalhan, C.T. Gillespie, S. Rosenberg, M. McAvoy DeCamp, A.S. Rogowski, J. Hixon, L.F. Angel, O. Dib, F.C. Sciurba, D. Chandra, M. Crespo, J. Bon Field, J. Rahul Tedrow, C. Ledezma, P. Consolaro, M. Beckner, A. Majid, G. Cheng, J. Cardenas‐Garcia, D. Beach, E. Folch, A. Agnew, W. Hori, A. Nathanson, M. Wahidi, S. Shofer, M. Hartwig, K. Mahmood, E. Smathers, W. Tillis, K. Verma, D. Taneja, M. Peil, S. Chittivelu, P. Doloszycki, P.E. Whitten, B. Aulakh, O. Ikadios, J. Michel, J. Crabb, B. McVay, A. Scott, E.A. Pautler, T.A. Connolly, J.F. Santacruz, L. Kopas, R. Parham, B. Solid, W. Krimsky, F. Gregoire, S. King, A. Mehta, F. Almeida, T. Gildea, J. Cicenia, M. Machuzak, S. Sethi, Y.M. Meli, J. Baran, R. Rice, D. Faile, N. Rai, K. Jensen, R. Kahlstrom, A. Haroon, R. Ionita, F. White, D. Watkins, B. Moore, H. Soukiasian, H. Merry, Z. Mosenifar, S. Ghandehari, D. Balfe, J. Park, R. Mardirosian, J.S. Ferguson, J. Kanne, D. Sonetti, D. Modi, M. Regan, J. Maloney, M. Hackbarth, M. Gilles, A. Harris, A. Maser, J.T. Puchalski, C. Rochester, J. Possick, K. Johnson, Z. Dabre, K. Kovitz, M. Joo, J. DeLisa, S.V. Villalan, G. Krishna, J. Canfield, A. Marfatia, E. Selley, S.V. Villalan, J. Utz, D. Midthun, R. Kern, E.S. Edell, L.L. Boras, S. Gay, K.A. Bauman, M. King Han, R.L. Sagana, K. Nelson, C. Meldrum, M. Jantz, H.J. Mehta, C. Eagan, J. West, A. Delage, S. Martel, P. LeBlanc, F. Maltais, Y. Lacasse, N. Lampron, F. Laberge, J. Milot, J. Picard, M.J. Breton, M. Dransfield, J.M. Wells, S. Bhatt, P. Smith, E.N. Seabron‐Harris, K. Hammond, C. Egidio

**Affiliations:** ^1^ Department of Pulmonary diseases, University Medical Center Groningen University of Groningen Groningen The Netherlands; ^2^ Royal Brompton and Harefield NHS Trust Chelsea and Westminster Hospital and Imperial College London UK; ^3^ Department of medicine, division of Pulmonary, Allergy and Critical Care Medicine University of Pittsburgh School of Medicine Pittsburgh PA USA; ^4^ Thoraxklinik and Translational Lung Research Center (TLRC) University of Heidelberg Heidelberg Germany

**Keywords:** bronchoscopy and interventional techniques, emphysema, endobronchial coils, lung volume reduction, quantitative computed tomographic analysis

## Abstract

**Background and objective:**

The RENEW trial demonstrated that bronchoscopic lung volume reduction using endobronchial coils improves quality of life, pulmonary function and exercise performance. In this post hoc analysis of RENEW, we examine the mechanism of action of endobronchial coils that drives improvement in clinical outcomes.

**Methods:**

A total of 78 patients from the RENEW coil‐treated group who were treated in one or both lobes that were deemed as the most destroyed were included in this retrospective analysis. Expiratory and inspiratory HRCT scans were used to assess lobar volume change from baseline to 12 months post coil treatment in treated and untreated lobes.

**Results:**

Reduction in lobar RV in treated lobes was significantly associated with favourable clinical improvement. Independent predictor of the change in RV and FEV_1_ was the change in lobar RV reduction in the treated lobes and for change in 6MWD the absence of cardiac disease and the change in SGRQ, while the independent predictor of change in SGRQ was the change in 6MWD.

**Conclusion:**

Our results suggest that residual lobar volume reduction in treated lobes measured by QCT is the driving mechanism of action of endobronchial coils leading to positive clinical outcomes. However, the improvement in exercise capacity and quality of life seems to be affected by the presence of cardiac disease.

## Abstract 2

Residual lobar volume reduction in treated lobes measured by QCT was found to be the driving mechanism of action of endobronchial coils leading to positive clinical outcomes. However, the improvement in exercise capacity and quality of life seems to be affected by the presence of cardiac disease.

**See related** Editorial

## INTRODUCTION

In patients with severe emphysema, severe lung hyperinflation impairs breathing mechanics, drastically diminishing quality of life and exercise tolerance. Current lung volume reduction techniques such as lung volume reduction surgery (LVRS) and endobronchial valves typically remove or collapse the most diseased regions to reduce hyperinflation.^1^ However, bronchoscopic lung volume reduction using shape‐memory nitinol endobronchial coils is a non‐blocking technology, in that it allows ventilation of the treated lobe.^2^ Mechanistically, endobronchial coils are presumed to be different from either traditional LVRS or endobronchial valves, although further investigation is needed to fully elucidate the mechanism of action of coils. Currently, only small studies have been performed to investigate the coil mechanism of action.^3^, ^4^, ^5^, ^6^

The RENEW trial, an international, multicentre randomized controlled trial, assessed endobronchial coil treatment in patients with severe lung hyperinflation and homogeneous or heterogeneous emphysema.^7^ In this post hoc analysis of RENEW, our aim was to examine the mechanism of action of endobronchial coils that drives improvement in clinical outcomes. We examined computed tomography (CT) scans obtained from RENEW to measure structural changes from baseline to 12 months to explore mechanistic insights on endobronchial coils.

## METHODS

### Patient population

The patients' characteristics and primary results of the RENEW trial (NCT01608490) have previously been reported.^7^ This was a retrospective analysis in which we included patients who participated in the RENEW trial and who were treated with coils bilaterally and had evaluable inspiratory and expiratory CT scans at baseline and 12‐month follow‐up (FU). Furthermore, patients were excluded when they were treated in one or both lobes that were not deemed as the most destructed when analysed by quantitative CT (QCT) analysis. As implanting coils in a lobe of lesser ipsilateral destruction may result in a differing mechanism of action, these patients were excluded from this mechanistic analysis.^8^, ^9^ The RENEW trial was approved by all the 31 trial sites' medical ethical review committees, and all patients provided written informed consent.

### QCT analysis

After the RENEW 12‐month primary endpoint FU visit and study un‐blinding, both baseline and 12‐month HRCT scans for the coil‐treated group were analysed quantitatively (QCT) (Thirona, Nijmegen, The Netherlands). The QCT analysis included volumetric and densitometry assessment at a lung and lobar basis. Percentage heterogeneity was calculated as difference in %LAA950 between ipsilateral lobes. Lobar volume change was calculated as the change in lobar volume from baseline to 12 months post coil treatment for each of the five lobes, assessed both with expiratory scans (lobar residual volume (RV) change) and inspiratory scans (lobar total lung capacity (TLC) change). Lobar volume change of the treated lobes was defined as the sum of lobar volume change of both treated lobes. Lobar volume change of the untreated lobes was defined as the sum of lobar volume change of the remaining three untreated lobes. For each lobe, lobar vital capacity (VC) was calculated as the difference between lobar RV and lobar TLC. Furthermore, patients performed 6‐min walk test (6MWT),^10^ lung function tests (spirometry and body plethysmography^11^, ^12^) and the St George's Respiratory Questionnaire (SGRQ)^13^ at baseline and after 12 months FU.

### Statistical analysis

A paired t‐test was performed to evaluate the difference between baseline and 12‐month FU in lobar volumes on CT and other clinical outcomes. Pearson correlation coefficients were calculated to evaluate the associations between QCT measured outcomes and clinical response outcomes. It was also utilized to evaluate the associations between change in lobar RV and lobar TLC volumes measured on CT. An independent t‐test was performed to evaluate the difference in change in clinical outcomes between patients with and without reduction in lobar RV in treated lobes. A linear multiple regression analyses (method enter) was performed to evaluate the independent predictors of change in the following clinical outcomes: forced expiratory volume in 1 s (FEV_1_), RV, 6‐min walk distance (6MWD) and SGRQ total score. To evaluate whether there is a difference in clinical outcome between two potential mechanisms of action, we performed an independent sample t‐test to evaluate the difference between patients with and without compensatory expansion of the untreated lobes. Compensatory expansion of the untreated lobes was defined as a change in lobar TLC volume in the untreated lobe of higher than 0 mL. All statistical analyses were performed using SPSS statistics version 23 (IBM, Armonk, NY, USA). *P*‐values of <0.05 were considered statistically significant.

## RESULTS

Of the 158 patients randomized to the RENEW treatment group, 125 completed bilateral treatment and had evaluable inspiratory and expiratory HRCT scans at baseline and 12‐month FU. Of these 125 patients, 47 were treated in one or both lobes that were not deemed as the most destructed when analysed by QCT.^8^ In total, 78 patients were included in this analysis and baseline characteristics are shown in Table 1.

**Table 1:** Baseline characteristics (*n* = 78)

Age (years)	62.9 ± 8.1
Male, *n* (%)	37 (47%)
BMI (kg/m^2^)	24.9 ± 4.6
BODE index	6.1 ± 1.3
Number of comorbidities, *n*	2.7 ± 2.1
Four or more comorbidities, *n* (%)	25 (32%)
% Cardiac comorbidities	30.8 (24)
6MWD (m)	314 ± 76.9
RV (% predicted)	246.8 ± 40.6
RV/TLC (%)	67.1 ± 7.3
FEV_1_ (% predicted)	25.8 ± 6.4
SGRQ, total score	60.2 ± 13.6
Emphysema distribution†	
Heterogeneous, *n* (%)	22 (28%)
Homogeneous, *n* (%)	34 (44%)
Mixed, *n* (%)	22 (28%)
Emphysema (−950 HU) (%)	46.9 ± 12.9
Air trapping (−856 HU) (%)	77.7 ± 10.4

Data are presented as mean ± SD or number (%). Cardiac comorbidities were based on medical history reported by the investigator and included current coronary artery disease, congestive heart failure or atrial fibrillation, but in a stable situation (subjects were excluded when there was active/symptomatic CAD, CHF (LVEF < 45% on echocardiogram) or uncontrolled atrial fibrillation).

† Heterogeneous: ≥15% ipsilateral difference in %LAA950 in both lungs, homogeneous: <15% ipsilateral difference in %LAA950 in both lungs and mixed: one heterogeneous lung and one homogeneous lung.

%LAA950, % low‐attenuation area < −950 Hounsfield Unit; 6MWD, 6‐min walk distance; BMI, body mass index; BODE, BODE: combined index of B: Body mass index, O: Obstruction (FEV1), D: Dyspnea (mMRC) and E: exercise (6MWD); CAD, Coronary artery disease; CHF, Congestive heart failure; FEV_1_, forced expiratory volume in 1 s; HU, Hounsfield unit; LVEF, left ventricular ejection fraction; RV, residual volume; SGRQ, St George's Respiratory Questionnaire; TLC, total lung capacity.

The change in lobar volumes measured on CT and other clinical outcomes are shown in Table 2. Lobar RV and TLC significantly decreased after 12 months in the total lung and in treated lobes, while lobar RV and TLC in the untreated lobes significantly increased. Lobar VC did not change significantly. Furthermore, statistically significant clinical improvements were found in RV (body plethysmography), SGRQ total score and FEV_1_ but not in 6MWD.

**Table 2:** Change in lobar volumes on CT and other clinical variables between baseline and 12‐month FU (*n* = 78)

	Baseline	12‐month FU	Difference	*P*‐value
Lobar volumes on CT				
Change in lobar RV in total lung (mL)	5679 ± 1164	5450 ± 1238	−229 ± 659	**0.003**
Change in lobar RV in treated lobes (mL)	2899 ± 688	2563 ± 735	−337 ± 467	**<0.001**
Change in lobar RV in untreated lobes (mL)	2780 ± 708	2888 ± 758	108 ± 409	**0.022**
Change in lobar TLC in total lung (mL)	7018 ± 1230	6850 ± 1223	−168 ± 427	**0.001**
Change in lobar TLC in treated lobes (mL)	3449 ± 730	3189 ± 767	−259 ± 354	**<0.001**
Change in lobar TLC in untreated lobes (mL)	3569 ± 761	3660 ± 797	92 ± 372	0.033
Change in lobar VC in total lung (mL)	1339 ± 622	1399 ± 716	61 ± 686	0.437
Change in lobar VC in treated lobes (mL)	549 ± 275	627 ± 343	77 ± 360	0.62
Change in lobar VC in untreated lobes (mL)	789 ± 406	773 ± 503	−17 ± 441	0.741
Change in clinical variables				
Change in RV, body box (mL)	5282 ± 1075	4844 ± 1202	437 ± 897	**<0.001**
Change in 6MWD (m)	314 ± 77	322 ± 105	7.5 ± 74	0.376
Change in SGRQ, total score	60.2 ± 13.6	51.1 ± 16.2	−9.1 ± 12.9	**<0.001**
Change in FEV_1_ (mL)	709 ± 190	770 ± 231	60.4 ± 152	**0.001**

Data are presented as mean ± SD. Paired t‐test was performed to evaluate the difference between baseline and 12‐month FU. Significant *P*‐values are depicted in bold.

6MWD, 6‐min walk distance; CT, computed tomography; FEV_1_, forced expiratory volume in 1 s; FU, follow‐up; RV, residual volume; SGRQ, St George's Respiratory Questionnaire; TLC, total lung capacity; VC, vital capacity.

The association between QCT measured outcomes and clinical response outcomes are shown in Table 3. The change in lobar RV in the treated lobes was significantly associated with favourable clinical improvements in RV, FEV_1_ and 6MWD. Change in lobar RV on CT showed the strongest correlations to clinical outcomes across all QCT measures analysed.

**Table 3:** Associations between change in QCT‐measured outcomes and change in clinical response variables

		Lobar RV change			Lobar TLC change		
Parameter		Treated lobes	Untreated lobes	Total lung	Treated lobes	Untreated lobes	Total lung
Change in RV (*n* = 78)		0.380 (**0.001**)	−0.168 (0.142)	0.165 (0.148)	0.322 (**0.004)**	−0.250 (**0.027)**	0.049 (0.670)
Change in FEV_1_ (*n* = 78)		−0.706 (**<0.001**)	0.088 (0.442)	−0.446 (**<0.001**)	−0.454 (**<0.001**)	0.481 (**<0.001**)	−0.042 (0.712)
Change in SGRQ (*n* = 78)		0.167 (0.144)	−0.256 (**0.023)**	−0.041 (0.724)	0.169 (0.140)	−0.426 (**<0.001**)	−0.231 (**0.042)**
Change in 6MWD (*n* = 77)		−0.232 (**0.042**)	0.005 (0.966)	−0.162 (0.160)	−0.111 (0.338)	0.168 (0.145)	0.055 (0.636)
Change in lobar TLC (*n* = 77)	Treated lobes	0.647 **(<0.001)**	−0.336 **(0.003)**	0.242 **(0.033)**			
	Untreated lobes	−0.212 (0.062)	0.364 **(0.001)**	0.142 (0.216)			
	All lobes	0.327 **(0.004)**	−0.012 (0.915)	0.260 **(0.022)**			

Data are presented as Pearson correlation coefficient (*P*‐value). Significant *P*‐values are depicted in bold.

6MWD, 6‐min walk distance; FEV_1_, forced expiratory volume in 1 s; QCT, quantitative computed tomography; RV, residual volume; SGRQ, St George's Respiratory Questionnaire; TLC, total lung capacity.

While Table 3 showed quite strong correlations between lobar RV reduction in the treated lobes and RV and FEV_1_, the correlations to the functional endpoints of 6MWD and SGRQ were weaker or non‐significant (possibly due to the lower number of subjects and hence inadequate power). Furthermore, patients with reduction in lobar RV in the treated lobes (>0 mL) significantly improved in RV and FEV_1_ but not in SGRQ and 6MWD compared to patients with no lobar RV reduction in treated lobes (Table S1 in Supplementary Information). We performed a multiple linear regression model to investigate what drives the changes in the different clinical endpoints. Table S2 (Supplementary Information) shows that the independent predictors of change in 6MWD are the absence of cardiac disease and the change in SGRQ, while the independent predictor of change in SGRQ is the change in 6MWD. The independent predictor of both the change in RV and FEV_1_ is the change in lobar RV reduction in the treated lobes.

**Figure 1:**
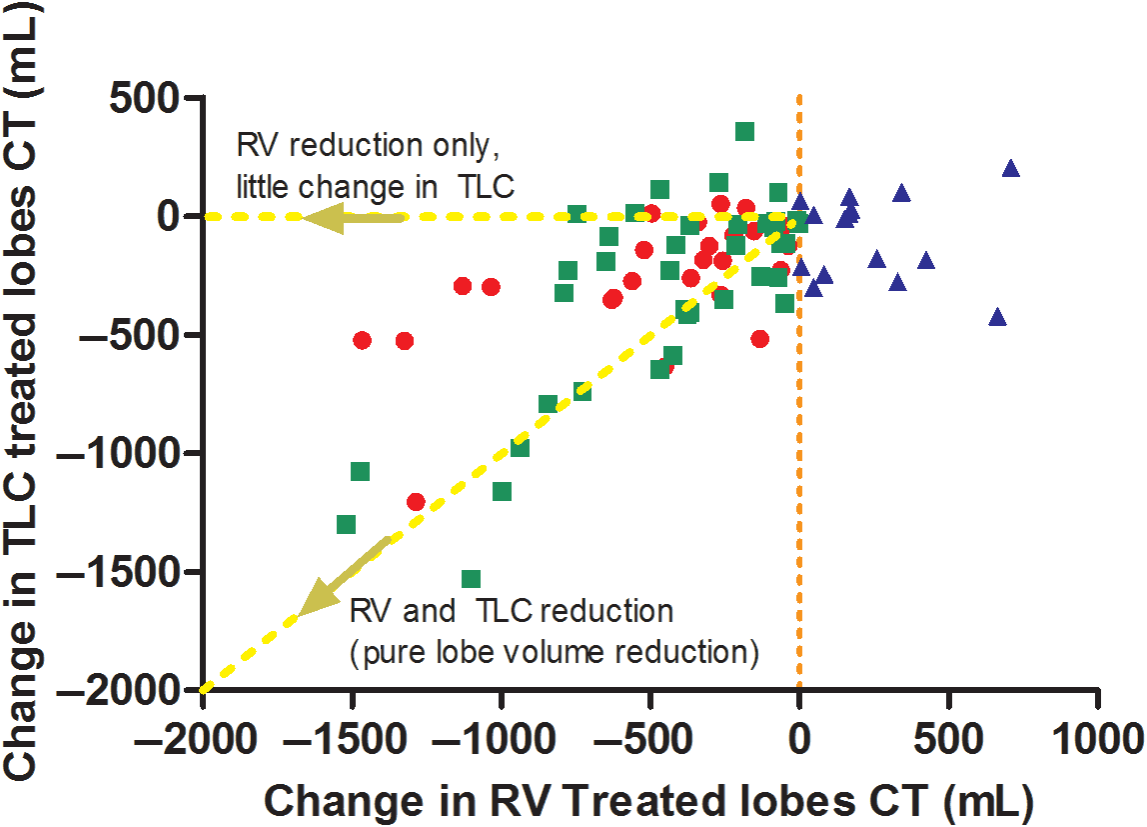
Scatterplot of change in TLC in treated lobes and change in RV in treated lobes, divided by difference in groups with different mechanisms of action and non‐responders. 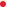, Responders without compensatory expansion; 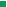, responders with compensatory expansion; 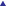, non‐responders (no change in lobar RV). CT, computed tomography; RV, residual volume; TLC, total lung capacity.

Two different mechanisms of action can occur in the patients with reduction in lobar RV in the coil treated lobes. One group shows compensatory expansion of the untreated lobes and one does not show compensatory expansion of the untreated lobes (Table S3 (Supplementary Information), Fig. 1). Except for SGRQ total score, groups with or without compensatory expansion did not significantly differ in change in clinical outcomes.

## DISCUSSION

Emphysema is characterized by loss of the lung's natural elastic recoil, which causes unsupported airways to collapse during exhalation. This, in turn, causes air trapping and increased lung volume, which makes breathing difficult. Endobronchial coils have been designed to treat this specific pathophysiological challenge by compressing lung parenchyma, which in turn creates tissue tension and restores radial support, thereby tethering airways open to reduce airway collapse and air trapping.^2^, ^14^, ^15^ This is independent of collateral ventilation. While the design intent of the coils is well documented, the structural changes resulting from coil treatment have not previously been quantified. In this context, the reduction in hyperinflation measured at a lobar level has never been clearly demonstrated.

Spirometry and body plethysmography techniques measure whole lung volumes, while differential effects in treated versus untreated lobes must be expected after coil treatment. In a small cohort of 18 patients, it had been shown for the first time that the lung volume reduction coil treatment reduces the volume and the emphysema score at the treated lobes.^3^ In our analysis of 78 RENEW patients, we confirm that coils significantly reduce lobar RV in the treated lobes, and this is strongly correlated with changes in lung function. Thus, lobar RV reduction in the treated lobes could be an important structural change driving clinical improvements.

**Figure 2:**
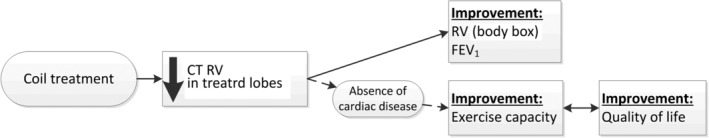
Scheme of potential mechanism of action of the coil treatment and effect on clinical outcomes. CT, computed tomography; FEV_1_, forced expiratory volume in 1 s; RV, residual volume.

**Figure 3:**
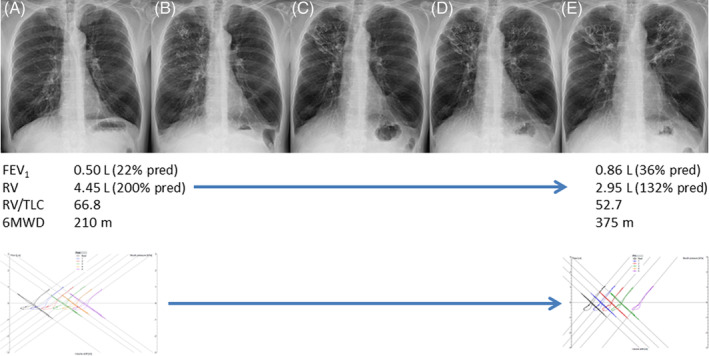
Example of mechanism of action of the endobronchial coil treatment in a patient. (A) Pre‐treatment X‐ray, (B) just post treatment showing coils in position in the right upper lobe, (C) 6 weeks post right upper lobe treatment showing additional visible volume reduction of this lobe, (D) just post‐treatment left upper lobe, (E) 6 weeks post left upper lobe treatment also showing additional visible volume reduction of this lobe. Below the images, the baseline and follow‐up efficacy parameters are given for this patient. All pre‐ and post‐treatment pressure–volume curves are given at the end, showing a much more efficient expiratory loop. 6MWD, 6‐min walk distance; FEV_1_, forced expiratory volume in 1 s; RV, residual volume; TLC, total lung capacity.

However, in contrast to lung function parameters, the change in lobar RV reduction in the treated lobes was less significantly associated with a change in exercise capacity or quality of life. The RENEW primary analysis already showed that comorbidities substantially influence the patient's improvement in 6MWT and SGRQ.^7^ In line, our results show that the independent predictors of improvement in 6MWD are the absence of cardiac disease and the change in quality of life. Furthermore, the independent predictor of an improvement in quality of life was the change in 6MWD. Therefore, it seems that the improvement in exercise capacity and quality of life is not driven by the change in lobar RV reduction, but is dependent of the presence or absence of cardiac disease and the change in quality of life or exercise capacity (Fig. 2). However, as the reduction in lung volume is less compared with, for example, LVRS or the treatment with endobronchial valves, it could also be that this leads to a less significant association between the change in lung volume and exercise capacity and quality of life and that cardiac comorbidities have more impact. More profound lung volume reduction has shown a positive effect on cardiac function,^16^, ^17^ which could mitigate the impact of cardiac comorbidities on the change in exercise capacity. Endobronchial valves have shown a substantial reduction in lobar TLC due to the induced full lobar collapse^18^ and volume changes on inspiratory CT scans post‐treatment are used to establish treatment effectiveness.^19^, ^20^ It also seems that the targeted lung volume reduction for valves is much larger when compared to coils, also indicating a different mechanism of action. While valves show substantial reductions in lobar TLC, our results show that endobronchial coils instead show a reduction in lobar RV accompanied by a modest reduction in lobar TLC. We divided our patients with a reduction in lobar RV into two groups: one group with a mild compensatory expansion of the untreated lobes was observed and one group without any compensatory expansion, as these could be different mechanisms of action. These groups could represent two different mechanisms of action of the coil treatment (Fig. 1): (i) restoration of elastic recoil and (ii) classic lung volume reduction. The first potential mechanism of action includes patients *without* compensatory expansion of untreated lobes. In these patients, the RV reduces and TLC does not and therefore VC increases. Therefore, the change in clinical benefit could be caused by decrease in air trapping in treated and/or untreated lobes which could increase the elastic recoil. The second potential mechanism of action includes the patients *with* compensatory expansion. In these patients, both RV and TLC of the treated lobe are reduced which can be classified as ‘classic lobe volume reduction’. With the coil treatment, this could be caused by compression of the tissue but also by reactive changes like the so called ‘coil‐associated opacity’. The RENEW study showed that patients with coil‐associated opacities or pneumonia showed superior improvement in comparison with patient without.^7^ Figure 3 shows an example of these mechanisms of action of the coil treatment in a patient.

Clinical outcomes significantly improved in both the groups with or without compensatory expansion. There was no significant difference between the groups suggesting that neither mechanism is superior to the other, although there was an unexplained significant improvement in SGRQ in the group with compensatory expansion.

SGRQ also significantly improved in patients without lobar RV reduction in the treated lobes so could be caused by a placebo effect.

Due to the retrospective design, this study has some limitations which could have influenced the results. As the RENEW study was not designed to answer this specific question, the study might be underpowered and a prospective study with a larger sample size needs to be performed to confirm these results. Furthermore, we excluded patients who were treated in an incorrect lobe, due to the fact that this analysis was focused on mechanism of action. However, this could have led to a selection bias.

In conclusion, our results suggest that residual lobar volume reduction in treated lobes measured by QCT is potentially the most important mechanism of action of endobronchial coils leading to positive clinical outcomes. However, the improvement in exercise capacity and quality of life seems to be affected by the presence of concurrent cardiac disease. Furthermore, we investigated two potential different mechanisms of action: residual lobar volume reduction with compensatory expansion of the untreated lobes and without compensatory expansion. Both mechanisms resulted in clinical improvements and were equally effective.

## Author contributions

Conceptualization: P.L.S., F.S., F.J.F.H., D.‐J.S. Formal analysis: J.E.H., D.‐J.S. Investigation: P.L.S., F.S., F.J.F.H., D.‐J.S. Methodology: P.L.S., J.E.H., D.‐J.S Project administration: J.E.H. Writing—original draft: J.E.H., D.‐J.S. Writing—review and editing: P.L.S., F.S., F.J.F.H., D.‐J.S.

## Disclosure statement

The original RENEW study (NCT01608490) was supported by PneumRx, Inc., CA, USA. The current post hoc analysis involved statistical support from PneumRx, without any further financial support. P.L.S., F.J.F.H., F.S. and D.‐J.S. have been principal investigators of the original RENEW trial and advisors to PneuRx/BTG.

**Abbreviations**
%LAA950: % low‐attenuation area < −950 Hounsfield Unit
6MWD: 6‐min walk distance
6MWT: 6‐min walk test
CT: computed tomography
FEV_1_: forced expiratory volume in 1 s
FU: follow‐up
HRCT: high‐resolution CT
HU: Hounsfield unit
LVRS: lung volume reduction surgery
QCT: quantitative CT
RV: residual volume
SGRQ: St George's Respiratory Questionnaire
TLC: total lung capacity
VC: vital capacity

## Supporting information


**Table S1** Differences in change in clinical outcomes between patients with and without reduction in lobar RV in treated lobes.
**Table S2** Linear regression models with change in clinical outcomes as dependent variable.
**Table S3** Differences in change in clinical outcomes between groups with and without compensatory expansion of the untreated lobes.
