# Supplementary material for: Endobronchial coils for emphysema: Dual mechanism of action on lobar residual volume reduction
Source: Respirology. 2020 Apr 8;25(11):1160–6. doi: 10.1111/resp.13816 (PMC7687244; doi:10.1111/resp.13816)
Supplement: Supplementary file 1 — Table S1 Differences in change in clinical outcomes between patients with and without reduction in lobar RV in treated lobes. Table S2 Linear regression models with change in clinical outcomes as dependent variable. Table S3 Differences in change in clinical outcomes between groups with and without compensatory expansion of the untreated lobes. [file RESP-25-1160-s001.doc]

**SUPPLEMENTARY INFORMATION**

**Endobronchial Coils for emphysema: dual mechanism of action on lobar residual volume reduction**

Jorine E Hartman^1^, Pallav L Shah^2^, Frank Sciurba^3^, Felix JF Herth^4^ and Dirk-Jan Slebos^1^ *on behalf of the RENEW Study Group*

^1^Department of Pulmonary diseases, University of Groningen, University Medical Center Groningen, Groningen, The Netherlands.

^2^ Royal Brompton & Harefield NHS Trust, Chelsea & Westminster Hospital and Imperial College, London, United Kingdom.

^3^University of Pittsburgh School of Medicine, Pittsburgh, PA, USA

^4^Thoraxklinik and Translational Lung Research Center (TLRC), University of Heidelberg, Heidelberg, Germany

**Table S1.** Difference in change in clinical outcomes between patients with and without reduction in lobar RV in treated lobes

|  | **Change in Lobar RV volume treated lobes** | | |
| --- | --- | --- | --- |
|  | <0 mL (n=62) | >0mL (n=15) | p-value |
| **Change in 6MWD** | 13.76 | -18.4 | 0.131 |
| **Change in SGRQ** | -9.38 | -8.04 | 0.721 |
| **Change in RV** | -0.539 | -0.011 | **0.040** |
| **Change in FEV_1_** | 0.091 | -0.068 | **<0.001** |

Data are presented as mean change and p-value. Significant values (p<0.05) are depicted in bold.

Differences between groups we calculated with an independent sample t-test.

RV: Residual Volume, mL: milliliter, 6MWD: 6-minute walk distance, SGRQ: St. George’s Respiratory Questionnaire, FEV_1_: Forced Expiratory Volume in 1 second

**Table S2 Linear regression models with change in clinical outcomes as dependent variable.**

|  | **Change in 6MWD** | | **Change in SGRQ** | | **Change in FEV_1_** | | **Change in RV** | |
| --- | --- | --- | --- | --- | --- | --- | --- | --- |
|  | B | p-value | B | p-value | B | p-value | B | p-value |
| Change in lobar RV treated lobes | -9.87 | 0.655 | 0.599 | 0.885 | -0.219 | **<0.001** | 0.722 | **0.001** |
| Change in 6MWD |  |  | -0.078 | **<0.001** | <0.001 | 0.636 | <0.001 | 0.775 |
| Change in SGRQ total score | -2.21 | **<0.001** |  |  | -0.001 | 0.475 | 0.006 | 0.490 |
| Change in FEV_1_ | 29.16 | 0.699 | -6.61 | 0.639 |  |  |  |  |
| Change in RV | -0.98 | 0.920 | 0.79 | 0.666 |  |  |  |  |
| Comorbidity number ≥ 4 | -25.98 | 0.157 | -3.78 | 0.272 | -0.038 | 0.221 | -0.305 | 0.202 |
| Absence of cardiac disease | 37.09 | **0.042** | 1.33 | 0.701 | -0.015 | 0.643 | -0.015 | 0.950 |

Data are presented as B and p-value. All variables in the linear regression model (method enter) are shown. Significant values (p<0.05) are depicted in bold.

RV: Residual Volume, 6MWD: 6-minute walk distance, SGRQ: St. George’s Respiratory Questionnaire, FEV_1_: Forced Expiratory Volume in 1 second

**Table S3** Difference in change in clinical outcomes between groups with and without compensatory expansion of the untreated lobes.

|  | **Change in 6MWD** | **Change in SGRQ** | **Change in RV** | **Change in FEV_1_** |
| --- | --- | --- | --- | --- |
| Without compensatory expansion (n=25) | 16.3 ± 74.4 | -4.6 ± 10.2 | -0.59 ± 0.94 | 0.09 ± 0.12 |
| With compensatory expansion (n=37) | 12.1 ± 78.8 | -12.5 ± 14.0 | -0.50 ± 0.82 | 0.09 ± 0.16 |
|  |  |  |  |  |
| No change in lobar RV in treated lobes (n=15) | -18.4 ± 57.1 | -8.04 ± 12.3 | -0.01 ± 0.93 | -0.07 ± 0.11 |

Data are presented as mean ± standard deviation. Only change in SGRQ significantly (p<0.05) differed between the group with and without compensatory expansion (tested with an independent sample t-test).

Compensatory expansion: Change in TLC lobar volume in untreated lobes> 0mL.

RV: Residual Volume, 6MWD: 6-minute walk distance, SGRQ: St. George’s Respiratory Questionnaire, FEV_1_: Forced Expiratory Volume in 1 second
